# Accounting for unintended consequences of resource policy: Connecting research that addresses displacement of environmental impacts

**DOI:** 10.1111/conl.12628

**Published:** 2019-02-06

**Authors:** Rebecca L. Lewison, Andrew F. Johnson, Jianbang Gan, Robin Pelc, Katie Westfall, Mark Helvey

**Affiliations:** ^1^ Department of Biology San Diego State University San Diego California; ^2^ MarFishEco Portland Oregon; ^3^ Department of Ecosystem Science and Management Texas A&M University College Station Texas; ^4^ California State University Monterey Bay Seaside California; ^5^ Environmental Defense Fund New York New York; ^6^ NOAA Fisheries West Coast Region Long Beach California

**Keywords:** backfire, environmental load displacement, leakage, rebound, slippage, spillover, sustainability, transfer effects, unequal ecological exchange

## Abstract

Natural resource policies enacted to protect environmental integrity play an important role in promoting sustainability. However, when resources are shared ecologically, economically, or through a common, global interest, policies implemented to protect resource sustainability in one domain can displace, and in some cases magnify, environmental degradation to other domains. Although such displacement has been recognized as a fundamental challenge to environmental and conservation policy within some resource sectors, there has been little cross‐disciplinary and cross‐sectoral integration to address the problem. This suggests that siloed knowledge may be impeding widespread recognition of the ubiquity of displacement and the need for mitigation. Here, we connect research across multiple disciplines to promote a broader discussion and recognition of the processes and pathways that can lead to displaced impacts that countermand or undermine resource policy and outline a number of approaches that can mitigate displacement.

## 1 INTRODUCTION

The UN's 2030 Agenda for Sustainable Development (UN Sustainable Development Goals, n.d.) identifies the need for responsible and sustainable consumption and production as a key goal. Environmental and conservation policies, enacted to protect environmental integrity, play an important role in promoting this sustainability. However, in some cases, policies enacted to improve or protect environmental quality can backfire. When resources are shared or linked ecologically or economically through physical movement of resources (i.e., migration and trade) or through a common, global interest (e.g., carbon sequestration, species extinction, biodiversity conservation), policies in one jurisdiction can displace, and in some cases magnify, environmental degradation beyond a policy's intended boundaries. This is particularly evident in domains where environmental, conservation, or resource use governance is less stringent. Although this displacement often arises across geographic boundaries, the displacement can occur across many boundaries, for example, community, sectoral, or temporal boundaries, herein referred to as jurisdictions, and can impact entities ranging from regional or national governments, resource sectors, communities, or individual households (Aichele & Gelbermayr, 2015; Fargione, Hill, Tilman, Polasky, & Hawthorne, 2008; Oliveira et al., 2007).

The unintentional displacement or transfer of environmental impacts from one jurisdiction to another has been studied by disparate research disciplines through divergent epistemological lenses (Aukland, Costa, & Brown, 2003; Bunker, 1984; Friis et al., 2016; Meyfroidt, Lambin, Erb, & Hertel, 2013; Paltsev, 2001; Wu, 2000). Although within some resource sectors, processes and pathways that displace environmental impacts elsewhere are seen as a fundamental challenge to resource policy (Fargione et al., 2008; Oliveira et al., 2007), there has been little cross‐disciplinary or cross‐sectoral integration, suggesting that siloed approaches in exploring these processes and unintended outcomes may be impeding widespread recognition of their ubiquity. A compounding body of literature suggests that a failure to recognize and account for these outsourced effects can jeopardize or undermine the efficacy of environmental or conservation policy.

## 2 RECOGNIZING HOW ENVIRONMENTAL IMPACTS CAN BE DISPLACED

**Figure 1:**
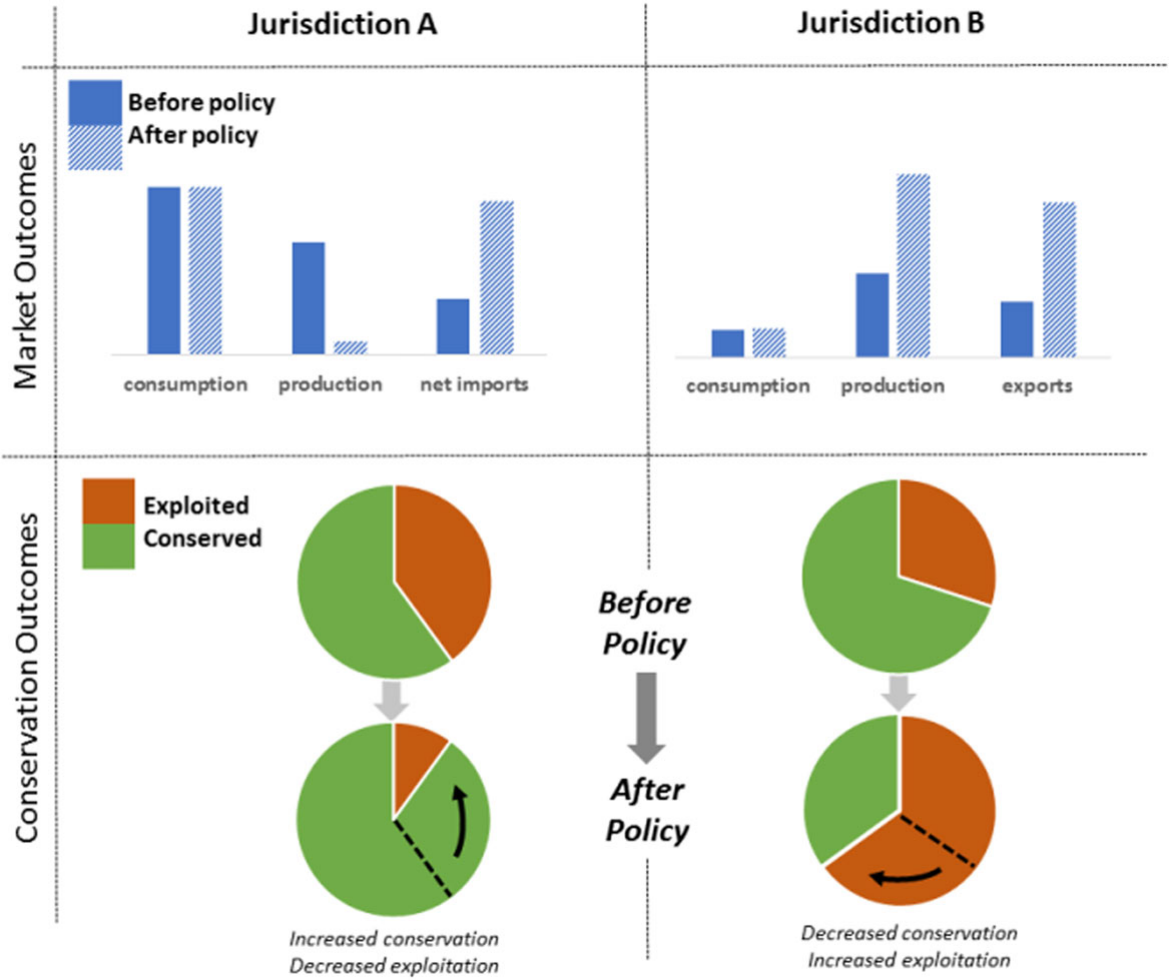
Diagrammatic representation of a negative displacement of environmental impacts. Here we illustrate a common scenario in which a policy designed to protect natural resources in one jurisdiction (A) improves local conservation outcomes but leads to reduced conservation outcomes in another jurisdiction (B) which, in many cases, can lead to a poorer conservation outcome overall. When production or extraction activities are curtailed in A due to environmental and conservation policies, consumption demands in A are met by increased imports from B. This results in a larger, negative environmental footprint or impact in B, which may occur when there is weak governance of resource use in B.

Policies designed to curtail or eliminate environmental degradation from production, extraction, or consumption activities within a particular jurisdiction (Sabatier, 1988) can impact other jurisdictions. Although in some cases displacement can lead to positive outcomes—a policy in one jurisdiction improves the conservation outcomes in others—there are many more documented examples of its negative impacts. In the case of negative displaced impacts, a conservation policy designed to improve environmental quality in one jurisdiction degrades conservation outcomes or environmental quality in another jurisdiction, often resulting in a “zero‐sum conservation game” (Hornborg, 2009) or worse (Searchinger et al., 2008) (Figure 1).

Persistent and unsustainable production, consumption, and regional or global trade are important drivers of displacement. Consumers may unknowingly contribute to transferred environmental impacts, creating the consumption–environmental degradation paradox (Jorgenson & Rice, 2005; Lim, Carrasco, McHardy, & Edwards, 2017) where consumption‐based ecological burdens are passed onto the producing jurisdiction. Given the complex connectivity of global markets and ecological systems, it is challenging to directly measure these transferred or displaced impacts. However, recent research has identified displaced environmental impact across a range of natural resource policies, including those governing fisheries management (Chan & Pan, 2016; Helvey, Pomeroy, Pradhan, Squires, & Stohs, 2017; Rausser, Hamilton, Kovach, & Stifter, 2009), biodiversity protection (Lenzen et al., 2012; Weinzettel, Hertwich, Peters, Steen‐Olsen, & Galli, 2013), forest preservation (Gan & McCarl, 2007; Mayer, Kauppi, Angelstam, Zhang, & Tikka, 2005; Meyfroidt & Lambin, 2009), and land use (Kastner, Kastner, & Nonhebel, 2011; Meyfroidt, 2017). Despite growing evidence (Box 1), there has been relatively little effort within and across resource sectors to adequately integrate this work into policy discussions in a manner that transcends disciplinary, sectoral, or other boundaries. This likely explains why the unintended displacement of environmental impacts and necessary solutions have yet to be widely incorporated into the design and evaluation of conservation or natural resource policy.

**Box 1. Growing evidence of displaced environmental impacts from a wide range of policy contexts and resource sectors**

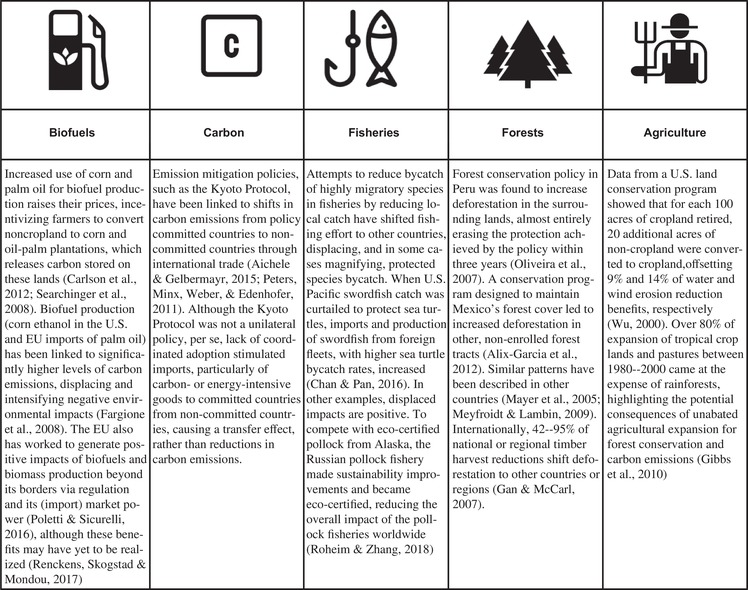

## 3 CONNECTING RESEARCH ACROSS DISCIPLINES

The displacement of environmental impacts has been well represented in research from a range of disciplines including economics, sociology, environmental policy, natural resource management and conservation, and environmental sciences (Alix‐Garcia et al, 2012; Aukland et al., 2003; Gellert, Frey, & Dahms, 2017; Hornborg, 2009; Searchinger et al., 2008). Disciplinary convergence has led to the development of many field‐specific terms and concepts that describe how resource use and extraction can lead to displacement and, in some cases, magnification, of environmental impacts across boundaries (Table 1). Economists use the broad term *externalities* to describe how one activity may affect the welfare of entities that are not the intended targets of the activity (Henderson, 1977), referred to as non‐target entities. *Leakage, slippage*, or *spillover* have also been used in economics research to describe how a policy can lead to displacement of intended economic or ecological outcomes from a target jurisdiction to non‐target jurisdictions (Paltsev, 2001; Rausser et al., 2009; Wu, 2000). The use of the term *spillover* in this context is distinct from how this term is used in the context of ecological protected areas, which uses the same term to describe increased production or recruitment of individuals in areas adjacent to no‐take zones (McClanahan & Mangi, 2000; Russ & Alcala, 2011). In natural resource management and resource policy domains, *land use displacement* and *indirect land use change*, in which market forces displace land use practices, are also terms used to describe the process of transferred impacts (Meyfroidt, 2017). Sociologists describe *unequal ecological exchange* to capture inequalities in trade‐governed resource use and extraction in which economically strong regions outsource their high‐consumption, natural resource demands to economically and environmentally weaker regions or countries, depleting their resources in the process (Jorgenson & Rice, 2005). The term *environmental load displacement* is used to describe consumption‐based indicators of anthropogenic pressure or “load” (e.g., ecological footprint) that are transferred elsewhere (Hornborg, 2009). In energy resource research, *rebound effect* and *backfire* have been used to describe how efficiency improvements influence energy consumption, a non‐intuitive effect whereby improved resource use efficiency increases rather than reduces overall resource use due to changes in market prices and economic growth (Gillingham, Rapson, & Wagner, 2016; Saunders, 1992). Finally, recent research in coupled human natural systems (Liu et al., 2007) has used the term *telecoupling* to describe reciprocal relationships in land use changes across disparate locations (Adger, Eakin, & Winkels, 2009) where feedbacks and multidirectional interactions occur among distant land use systems and *teleconnection* to emphasize how drivers of land system changes exert influence across distinct locations (Friis et al., 2016; Liu et al., 2013).

**Table 1:** Discipline‐specific terms and concepts that describe processes and pathways that can lead to displacement of environmental impacts across boundaries, with seminal examples from the literature

Terminology	Description	Key examples
Externality	An agent's activity affects the welfare of other agents who do not intend to bear the burden or receive the benefit (Pigou, 1920; Buchanan & Stubblebine, 1962)	Positive externalities with forest ecosystem services (Glück, 2000) Negative externalities with pollution (Henderson, 1977)
Leakage	When a policy action in a jurisdiction leads to the relocation or diffusion of some production and associated economic and environmental outcomes to other jurisdictions (Felder & Rutherford, 1993; Paltsev, 2001)	Carbon (Babiker 2005) Forest conservation (Gan & McCarl, 2007) Biodiversity conservation (Ewers & Rodrigues, 2008) Land use (Lambin & Meyfroidt, 2011)
Spillover effect	Used as a synonym for leakage and externality (Aukland et al., 2003; Buchanan & Stubblebine, 1962)	Timber (Wear & Murray, 2004) Land use policy (Hyde, Amacher, & Magrath, 1996)
Slippage effect	Often used as a synonym for leakage and indirect land use change (Leathers & Harrington, 2000; Wu, 2000)	Agricultural land conservation (Flemming, 2014) Forest conservation (Alix‐Garcia et al., 2012)
Indirect land use change/Land use displacement	Refers to the displacement of land use across spatial locations and/or sectors via the linkages of markets, often the prices and trade of commodities (Meyfroidt et al., 2013; Searchinger et al., 2008)	Corn ethanol production (Plevin, O'Hare, Jones, Torn, & Gibbs, 2010) Biofuel consumption (Overmars, Stehfest, Ros, & Prins, 2011) Biofuel production (Lapola et al., 2010) Deforestation (Meyfroidt & Lambin, 2009)
Unequal ecological exchange	Pioneered by Bunker (1984), this area of sociological inquiry theorizes the unequal material flows structured by trade and the corresponding movement of ecological footprints of economically strong regions to economically weaker ones (Gellert et al., 2017; Foster & Holleman, 2014)	Cocoa exports (Noble, 2017) Coffee trade (Austin, 2017) Deforestation (Jorgenson, Austin, & Dick, 2009)
Environmental load displacement	An area of sociological inquiry that theorizes on the economic and technological expansion of developed countries via foreign investment that occurs at the environmental expense of less‐developed nations (Jorgenson, 2016; Hornborg, 2008) creating a “zero‐sum game” model of sustainable development (Hornborg, 2009)	Carbon dioxide emissions (Grimes & Kentor, 2003) Water pollution (Jorgenson, 2009) Air pollution (Peng, Zhang, & Sun, 2016)
Rebound effect/backfire	Originally associated with the effect of energy use efficiency improvements on energy consumption (Jevons, 1866), this term suggests that an improvement in the use efficiency of a resource may not necessarily reduce the total use of the resource because the efficiency improvement will reduce the prices of the resource and its use and promote economic growth, thus stimulating consumption (Gillingham et al., 2016; Saunders, 1992)	Coal (Jevons, 1866) Electricity (Khazzoom, 1980) Gasoline (Small & van Dender, 2007) Irrigation (Dinar & Zilberman, 1991)
Teleconnection/telecoupling	Originates from atmospheric science to describe the linkages among climate anomalies over long spatial distances (Walker, 1923). Used to describe the connectivity of land use changes in different locations (Adger et al., 2009; Liu et al., 2013). Although teleconnection emphasizes the drivers of land system changes, telecoupling specifies feedback and multidirectional interactions among land use systems (Friis et al., 2016)	Climate systems (Bjerknes, 1969) Land use (Friis et al., 2016)

Although the concepts in Table 1 all relate to or describe displacement of impacts from one jurisdiction to another, they differ in the mechanisms, underlying drivers and responses or feedbacks that govern the individual processes and pathways, which can include ecological, economic, or social drivers. For example, externalities describe the welfare impact of one entity's activity on another when there is no market mechanism to counterbalance the external impact. The terms leakage, spillover, slippage, indirect land use change or displacement, and rebound effect result from responses by the impacted entity that negates expected environmental benefit primarily through economic drivers, for example, market value, price, or trade. However, the feedbacks from these processes and pathways may also differ. Because leakage, spillover, slippage, and land use change or displacement consider environmental impacts, their feedback mechanisms are primarily ecological ones. In contrast, feedback mechanisms for rebound effects are economic, that is, when a price reduction caused by an improvement in resource use, that is, increased efficiency, encourages more rather than less consumption of the resource. In teleconnection or telecoupling, linked climate changes across two distinct locations may be due to an ecological mechanism, whereas a coupled land use effect across two jurisdictions, similar to indirect land use change, is likely attributable to economic drivers. Likewise, in unequal ecological exchange and environmental load displacement, a particular economic activity (e.g., investment, technology expansion, trade) by an outside entity can lead to unwanted environmental, economic, or social impacts via multiple pathways in a country with weaker resource governance.

## 4 SOLUTIONS AND MITIGATION

Despite the ubiquity of displaced environmental impacts from a rich literature base that strives to characterize and understand the different forces that can undermine natural resource policy, there is still a need for stronger action and efforts to account for and mitigate displaced impacts that extend across disciplinary domains and resource sectors. There are a number of approaches and solutions that have been suggested to mitigate or avoid the unintended consequences of conservation policies that extend across disciplinary domains and resource sectors and recognize the complex mechanisms that influence sustainable production and consumption.

### 4.1 Explicitly consider displacement in policy design, scoping, and evaluation

Environmental resource policies must be framed or scoped within the appropriate social, economic, and cultural contexts at the relevant scale of the intended environmental change. This includes conducting analyses similar to reviews required by the U.S. National Environmental Policy Act to anticipate both direct and, just as importantly, indirect impacts. This formative review process can help decision‐makers evaluate tradeoffs and identify policy impacts on resource sourcing jurisdictions as well as provide additional measures needed to ensure resource extraction or use across other jurisdictions will not undermine intended goals. Such reviews require a comprehensive *ex ante* policy evaluation process that identifies broad environmental consequences (e.g., biodiversity impacts) from policy instruments that extend beyond a policy's target jurisdiction (Verones, Moran, Stadler, Kanemoto, & Wood, 2017).

### 4.2 Adopt multilateral landscape approaches

Landscape approaches have emerged as the most widely advocated means to address growing pressures on land, water, and other resources for accommodating environmental and biodiversity goals for present and future generations (Sayer et al., 2013). A multilateral landscape approach uses an adaptive rather than blueprint approach (Ostrom, 2007), recognizing the need to account for the diversity of resource stakeholders by using collaborative participation (Freeman, Duguma, & Minang, 2015), appreciating the multifunctional use of the same resource that covers structures, functions, and values (Selman, 2009), and understanding how outcomes on one scale are shaped by processes operating at other scales (Sayer et al., 2013). Although jurisdictions initiating a policy and the jurisdictions sourcing resources face different challenges in adopting a multilateral landscape approach, a policy that inherently recognizes the complex relationships among ecological, social, and economic systems and the influence these coupled relationships have on displaced impacts is essential (Kates et al., 2001; Sayer et al., 2013; Turner, Janetos, Verburg, & Murray, 2013) to support policy success. There are a number of current examples of how a resource management policy can backfire when a landscape perspective is not adopted, i.e., when a policy is adopted in one jurisdiction without coordination to adjacent or linked jurisdictions, it can lead to an overall increase in exploitation across the land or seascape (Cunningham, Bennear, & Smith 2016).

### 4.3 Enact both demand‐side and supply‐side policies

The displacement of environmental impacts (as shown in Figure 1) is more likely to stem from supply‐side policies, that is, policies that reduce supply in one jurisdiction, typically stimulating production in other jurisdictions. However, this response depends on the elasticity, or responsiveness to price change, of consumer demand. Elastic demand, in contrast to inelastic demand, is less likely to cause supply reduction in one jurisdiction to prompt production in another jurisdiction (Mukherjee, 2015).

One approach to reducing unintended displacement of environmental impacts is to enact policies or strategies that reduce demand for goods whose production generates negative environmental impacts in concert with supply‐side policies. By reducing demand, this approach ensures that the policies that reduce supply in one jurisdiction do not stimulate increased production or imports from other jurisdictions. Demand‐side policies can inform consumers of environmental consequences enabling them to make better buying decisions through the development of consumer guides, eco‐labels, or certifications. Eco‐certifications and other consumer‐facing programs create enhanced market access or price premiums for sustainable products, incentivize more sustainable production practices, and thus mitigate against unintended shifts of environmental impacts (Wu, 2013). For example, eco‐certification of sustainably produced pollock from Alaska has incentivized a fisheries improvement program for pollock fisheries in Russia, thereby leading to a net reduction in overall environmental impact from pollock production rather than displacement (Roheim & Zhang, 2018). Demand‐side interventions are also seen as critical to reducing deforestation (Walker, Patel, Davies, Milledge, & Hulse, 2013) and to sustainable development of alternative energy sources (Ji & Long, 2016).

To be effective, demand‐side approaches require traceability throughout the entire supply chain, matching the spatial scale of the market. Eco‐labels or certification programs that address a broad suite of sustainability goals rather than a single resource focus will help ensure there are not shifts to alternate products, which could also present challenges to sustainability. Innovative economic strategies that help finance conservation can enhance both the incentive and the economic capacity for producers to adopt more sustainable practices (Blackman & Rivera, 2011).

### 4.4 Reciprocity requirements for imports and trade agreements

Resource sector performance standards must be comparable among importing and exporting countries. For example, key U.S. resource policies (e.g., Magnuson‐Stevens Act; Marine Mammal Protection Act) allow the United States to prohibit imports from countries without stringent bycatch mitigation standards (Micheli et al., 2014; Williams, Burgess, Ashe, Gaines, & Reeves, 2016). However, financial support, capacity building, and guidance from importing countries are needed to promote sustainable conservation without exacerbating economic challenges for producers (Johnson et al., 2017; Williams et al., 2016) but access to funds and markets should remain contingent to meet environmental standards (Williams et al., 2016). Incorporating environmental stipulations into trade agreements can also be an important instrument to eliminate unfair competitive advantages for countries with less stringent environmental regulations (e.g., ending subsidies that contribute to the overexploitation of resources) and to achieve comparable levels of sustainability (George, 2014; Shandra, Leckband, McKinney, & London, 2009).

### 4.5 Enhance broad international cooperation

Broad cooperation among entities in the design and implementation of policy is needed to adopt and enforce complementary conservation policies to meet a shared conservation goal. A powerful analogue for this is the World Health Organization, which relies on broad international cooperation to combat disease (Hopkins, 2013). In the resource context, multinational resource management instruments like multilateral environmental agreements (MEAs), which require focused monitoring and compliance, will likely be an essential component to this cooperation. Another example of the importance of international collaboration to mitigate displaced impacts is the successful efforts to phase out chlorofluorocarbons (CFCs) globally. In 1987, 24 individual countries moved to reduce CFC production and consumption after CFC emissions were linked to ozone layer degradation, negotiating the Montreal Protocol. The Protocol, later ratified by all United Nations member countries, led to the first phase out of CFCs by developed countries by 1995 and the complete global phase out by 2010, with continued efforts to reduce other ozone‐depleting substances (UNEP Ozone Secretariat, n.d.). The scope of the international cooperation in these and other examples is key. Recent research suggests that displaced environmental impacts associated with forest conservation policies were generally reduced by broad international collaboration; however, limited cooperation, that is, cooperation among only a few countries, did not dramatically reduce the displacement of environmental impacts (Gan & McCarl, 2007).

## 5 CONCLUSIONS

Natural resource conservation policies will continue to be a fundamental tool for sustainable production and consumption. Although a number of disciplines have independently explored unintended cross‐boundary consequences of resource policies aimed to protect environmental integrity, the need to unify research across disciplines that relates to unintended shifts, displacement, or magnification of environmental impacts that can occur related to resource policy remains. Given how displaced environmental impacts can undermine conservation policies across a wide range of policy contexts, there is growing evidence that, to be effective in a global economy, policies must explicitly consider this broad range of processes in scoping, design, and evaluation. Without this explicit consideration, well‐intentioned conservation efforts may only create an illusion of resource preservation and conservation (Berlik, Kittredge, & Foster, 2002). While there is no simple fix, integrated approaches that draw from research across disciplines and resource sectors are needed. The use of demand‐side policies, import reciprocity requirements, trade regulations and agreements, multilateral landscape approaches, and broad international coordination can help ensure that conservation and resource use policies do not backfire and can actually have the intended, positive effect on environmental quality in both target and non‐target jurisdictions.
